# Carry-over in automatic analysers

**DOI:** 10.1155/S1463924684000201

**Published:** 1984

**Authors:** P. M. G. Broughton

**Affiliations:** Wolfson Research Laboratories Queen Elizabeth Medical Centre Birmingham B15 2TH UK


Journal of Automatic Chemistry, Volume 6, Number 2 (April–June 1984), pages 94–95

### Carry-over in automatic analysers

**P. M. G. Broughton**

*Wolfson Research Laboratories, Queen Elizabeth Medical Centre, Birmingham B15 2TH, UK*

### Introduction

It is now 20 years since the first description of sample carry-over or interaction in a continuous-flow analytical system [1]: that is, the error induced in the result of a specimen by contamination from the preceding one. Since then, the measurement of sample carry-over has been incorporated into several protocols for instrument evaluation and used as a criterion of instrumental performance [2–4], mainly for continuous-flow and discrete analysers. Recently, several new types of instrument have been introduced in which other forms of carry-over are possible. This paper considers methods of measuring these and discusses their effects on analytical precision.

### Sample carry-over

This may be measured by analysing two identical specimens with a high concentration of analyte (recorded as $a_1$ and $a_2$) followed by two identical specimens with a low concentration (which are recorded as $b_1$ and $b_2$). The carry-over ($k$) is usually [5] expressed as:

$$k = \frac{b_1 - b_2}{a_2 - b_2} \times 100\%.$$

Replicate measurements of $k$ are made, and the mean result should be the same for high–low and low–high sequences.

With most automatic analysers, carry-over is less than 1–2%, and usually this will not cause significant errors in routine analytical results. Consequently if the precision, measured using different sequences of specimens, is satisfactory, carry-over is unlikely to be significant and need not normally be measured as part of the evaluation of an instrument. If, however, the precision is poor, it may be necessary to test whether this is due to excessive carry-over.

When the two specimens have very different concentrations (for example $a = 500$, $b = 10$), a carry-over of 1% will produce an error of nearly 50% in the observed value $b_1$. In practice, however, one of these results (usually $a$) is likely to be outside the analytical range, and the specimen will need to be diluted and reanalysed. If the following specimen (with a value $b_1$) is also reanalysed, the original error due to carry-over will be obviated. Similarly when analysing a calibration standard or a blank, where a small carry-over error will be important, it is customary to analyse each specimen in duplicate and discard the first reading on each.

In some types of continuous-flow system, carry-over increases as the samples are pumped more rapidly through the system, and this may be a limitation to increasing the analytical rate. To compensate for this, it has been suggested [5] that if $k$ is large (for example 10%), the above equation should be used in an algorithm to correct the analytical results for carry-over. However, it is important to verify that $k$ is constant with time; if the correlation factor used differs from the true value at the time of analysis, errors will result.

Two other types of sample contamination have been postulated [2]: specimen cross-contamination arising from transfer of a portion of one specimen, via the sample probe, into the following one; and specimen-diluent contamination arising from contamination of a specimen by the diluent transferred from the probe of a sample-dilutor. However, neither of these effects seem to have been reported in instruments which have been evaluated.

### Reagent carry-over

Recently several new types of selective or random-access analyser have been introduced, in which different tests are performed in sequence on the same specimen. Other types of carry-over can then arise, which may be illustrated by considering two different adjacent specimens ($A$ and $B$) on which combinations of different tests ($R$, $S$, $T$ etc.) are performed in sequence.

Reagent carry-over could arise if reagent from one test ($R$) was carried over by the reagent probe into the reaction mixture of the following different test ($S$). The effect could, of course, be prevented by washing the probe between each reagent, but this would reduce the analytical rate. It is possible to test for gross reagent carry-over by using a concentrated dye solution for the first test and water for the second one [6]. However, it is not usually appropriate to express reagent carry-over as a volume, as with this method, because its importance depends on the nature of the reagents in the two tests. For example, contamination by a small volume of an enzyme inhibitor (for example cyanide) could have an adverse effect if the next test was an enzyme assay, or an assay depending on the use of an enzyme, but might have no effect on other types of test. Reagent carry-over is therefore best considered as the percentage error in test $S$ when it is preceded by test $R$. This principle has been used in testing one random-access analyser [7], in which a saturated solution of copper sulphate was used as the first test and a serum creatine kinase assay for the second. Any significant carry-over of copper ions would inhibit the creatine kinase. In this instance, no inhibition was observed, but this does not preclude the possibility that carry-over of smaller volumes of other reagents might affect other tests. Moreover, if an effect had been observed with this reagent combination, it might not be significant with other combinations.

A more comprehensive method of measuring reagent carry-over is to analyse a sequence of duplicate tests on the same specimen: $R_1$, $R_2$, $S_1$, $S_2$, $R_3$, $R_4$, etc. The error in test $S_1$ arising from carryover of reagent $R$ is then:

$$\frac{S_1 - S_2}{S_2} \times 100\%$$

and the error in test $R_3$, due to carry-over of reagent $S$, is

$$\frac{R_3 - R_4}{R_4} \times 100\%.$$

The specimen used should have a mid-range concentration of the analytes concerned, so that both inhibition and enhancement effects can be detected. Replicate measurements should be made for each sequence.

Since differences between duplicate tests (for example $S_1 - S_2$) could be due to random error, reagent carry-over should be considered significant only if replicate differences for a given sequence are all in the same direction (i.e. all positive or all negative), and if all differences are greater than twice the within-batch coefficient of variation (CV) for that concentration of analyte.

With many selective analysers, it is possible to select any sequence of a range of tests: with 12 tests, there are 132 possible sequences of pairs (for example RS, ST, SR, RT, TS, TR, etc.), and testing all of these would be tedious. However, since the effect of this type of carry-over is difficult to predict, the tests should be as comprehensive as possible.

### *Reagent-to-specimen carry-over*

This could arise if the sample probe came into contact with the reaction mixture before moving to aspirate the next sample. The effect on that specimen would again depend on the nature of the reagent and also on the volume of specimen in the cup, but when several tests are performed in sequence on the same specimen, the effect could be cumulative. One method used for measuring this type of carry-over [6] is with a dye as reagent and water as sample. If any dye is detected in the water sample, the effect of this carry-over with the reagents used routinely should be investigated.

### *Sample carry-over*

This could occur if a portion of the preceding sample was transferred by the sample probe into the reaction mixture of the next one. This can be tested by the conventional method described above, but in practice the effect might be greater if a test using a large sample volume was followed by a different one using a small volume [6]. This would be difficult to measure without including reagent carry-over, and it might then be necessary to use a radioisotope or dye solution as sample and to test whether this contaminated the following water blank.

So far, there have been no published reports that these types of carry-over occur in practice with selective analysers, although one manufacturer claims to have prevented them by using an immiscible non-reactive fluid as a barrier between the sample and reagents [6]. If reagent carry-over is shown to be significant for a particular sequence of tests, it would be advisable to avoid that sequence when arranging the order in which tests are to be performed routinely.

### Effect of carry-over on measurement of imprecision

These considerations become important when designing experiments to measure imprecision in selective analysers. Different specimen and test sequences can be used to include only sample carry-over, only reagent carry-over, neither, or both.

Usually [4], within-batch imprecision is determined by analysing a sequence of replicate samples for the same test: i.e. $A_1S$, $A_2S$, $A_3S$, etc. This sequence represents the optimal arrangement, where there are no effects from carry-over of sample or reagent. However, when measuring between-day or between-batch imprecision it is preferable for both successive tests and successive samples to be different so that the effects of both reagent and sample carry-over are included. This arrangement will then include all the carry-over errors which might be expected to occur in the analysis of a batch of patients' specimens. To do this comprehensively would require a complex design, in which specimens of three different concentrations [4] were analysed for possibly 12 different tests, in a different sequence on about 20 occasions. To cover all possible sequences of tests could take much longer. A more pragmatic approach is to randomize specimen and test sequences in order to obtain the 20 replicate results necessary for reliable calculation of the CV. Any test sequence which has previously been shown to be affected by reagent carry-over should be excluded.

### Conclusions

As new types of analyser are developed other types of carry-over will no doubt arise. It is important to recognize where these may occur, so that appropriate methods of testing for them can be devised, and to include these potential sources of error in designing experiments to measure imprecision.

### Acknowledgements

The author acknowledges financial support by the Department of Health and Social Security.

### References

1. Thiers, R. E. and Oglesby, K. M., *Clinical Chemistry*, **10** (1964), 246.
2. Broughton, P. M. G., Buttolph, M. A., Gowenlock, A. H., Neill, D. W. and Skentelbery, R. G., *Journal of Clinical Pathology*, **22** (1969), 278.
3. Young, D. S. and Gochman, N., *Standard Methods in Clinical Chemistry*, **7** (1972), 293.
4. Broughton, P. M. G., Gowenlock, A. H., McCormack, J. J. and Neill, D. W., *Annals of Clinical Biochemistry*, **11** (1974), 207.
5. Dixon, K., *Annals of Clinical Biochemistry*, **19** (1982), 224.
6. Smith, J., Svenjak, D., Turrell, J. and Vlastelica, D., *Clinical Chemistry*, **28** (1982), 1867.
7. Steindel, S. J. and Schoudt, P., *Journal of Clinical Laboratory Automation*, **3** (1983), 319.

### HARMONIZATION OF COLLABORATIVE ANALYTICAL STUDIES

The Second International Harmonization Symposium will take place at the National Academy of Sciences in Washington, D.C., 25–27 October 1984 and will be sponsored jointly by the Analytical Chemistry Division, the Applied Chemistry Division and the Clinical Chemistry Division of the International Union of Pure & Applied Chemistry and the Association of Official Analytical Chemists. The meeting is co-sponsored by the US National Committee for IUPAC and is also held in conjunction with the AOAC Centennial Annual International Meeting (running at the Shoreham Hotel during the following week).

There is no general agreement among organizations as to what constitutes an adequate design for a collaborative study to establish the performance of a method of analysis. From the results obtained under a specific collaborative study design, estimates are made of various components of variability. The symposium will discuss the design considerations used by organizations and will attempt to harmonize the requirements for an inter-laboratory study in terms of the number and nature of samples, laboratories and replicates, which will permit most groups to interpret the results for their particular purposes.

The first symposium was held in Helsinki in 1981: the proceedings were published early in the following year (*Collaborative Inter-laboratory Studies in Chemical Analysis* by H. Egan and T. S. West, Pergamon Press).

It is expected that representatives of the principal international agencies which organize collaborative analytical studies will take part, including the ISO, together with representatives of the sponsoring divisions of IUPAC and of the AOAC and certain national organizations.

*More information from Mrs K. Fominaya, Association of Official Analytical Chemists, Suite 210, 1111 N. 19th Street, Arlington, Virginia 22209, USA.*

